# Mad River Valley Dental Society

**Published:** 1862-04

**Authors:** 


### MAD RIVER VALLEY DENTAL SOCIETY.

THE Mad River Valley Dental Association met pursuant to adjournment, in the city of Dayton, on Tuesday evening, April 1st, in the office of Dr. W. A. Pease. Members present were Drs. Bradley, Willard, and Pease, of Dayton; J. H. Payne, of Franklin, O.; and Watt and Paine, of Xenia. The meeting, though small, was pleasant and profitable, and was highly edified by the reading of an Essay prepared by Dr. Pease on the use of Arsenic. We are glad to state that the Doctor, in accordance with the wishes of all present, has kindly consented to forward a copy of this able essay to the Dental Register for publication. We venture the prediction that it will be read with more interest and profit by the profession than any paper ever published on the same subject.

The Doctor, by the way, is not only an ornament to the profession, as an efficient and accomplished operator, but is a powerful auxiliary through his writings, in disseminating sound principles. May his example find many imitators. After an interesting, interlocutory discussion of some points in the Doctor's essay, the Society adjourned at midnight, to meet again in Dayton on the first Tuesday in July next.

PAINE.

